# Executive function assessment in New Zealand 2-year olds born at risk of neonatal hypoglycemia

**DOI:** 10.1371/journal.pone.0188158

**Published:** 2017-11-22

**Authors:** Judith M. Ansell, Trecia A. Wouldes, Jane E. Harding, on behalf of the CHYLD Study group

**Affiliations:** 1 Liggins Institute, University of Auckland, Auckland, New Zealand; 2 Department of Psychological Medicine, University of Auckland, Auckland, New Zealand; Boston Children's Hospital / Harvard Medical School, UNITED STATES

## Abstract

A growing number of babies are born with perinatal risk factors that may impair later development. These children are often assessed at 2 years to help predict outcome and direct support services. Executive function is an important predictor of academic achievement and behavior, but there are limited assessments of executive function in 2-year-olds and few have been tested in at-risk populations. Therefore, we developed a battery of four age-appropriate tasks to assess executive function in 2-year-olds. At 24 months’ corrected age 368 children completed tasks assessing attention, inhibition, working memory and cognitive flexibility. Scores on different tasks were weakly correlated, suggesting that they measured separate aspects of executive function, with combined scores for this cohort approximating a normal distribution. Significantly more boys (67%) than girls (57%) were unable to inhibit their behavior on the Snack Delay Task and girls (M = 3.24, SD = 2.4) had higher mean scores than boys (M = 2.7, SD = 2.7) on the Ducks and Buckets Reverse Categorization Task of working memory. Performance was significantly affected by family socioeconomic status. Mean scores were lower on all four individual tasks and on the global score of overall performance in children from a low household income (<$40,000) compared to those from medium ($40,001-$70,000) and high income households (>$70,001). Maternal education was only associated with scores on the working memory task and the global score; and a measure of neighborhood deprivation was only associated with scores on the two inhibitory tasks and the global score. Our findings confirm the feasibility of assessing executive function in 2-year-olds, and its ability to discriminate effects of socioeconomic status, a common confounder in child development research. Further development and standardization of this test battery comparing at-risk children with a normative population would provide a much-needed measure of executive function in early childhood.

## Introduction

Preschool executive function (EF) has been shown to be a better predictor of school readiness than either IQ or academic progress [1] and is positively associated with behavior [2, 3], mathematics [4, 5], reading [6] and overall achievement in older pre-school and school-age children [7, 8]. EF is a collective term for “higher order, self-regulatory processes that aid in the monitoring and control of thought and action” [9]. These processes include inhibitory control, (self-control, self-regulation), working memory and planning, and cognitive flexibility [10, 11]. Error correction and detection, resistance to interference and attentional control are also included in some definitions [2, 9, 10], with attention included here because of it’s fundamental importance for the development of EF overall.

EF begins to develop in the first few years [9] in a sequence corresponding to brain maturation, particularly in association with the prefrontal cortex [12]. Attention and inhibition are the earliest to emerge and underlie the later development of working memory and cognitive flexibility, although debate continues as to whether EFs are integrated but separate [2] or a singular construct in childhood [13]. This developmental sequence results in infants as young as 8 months being able to display simple inhibition of a prepotent response [14], with children at 3–4 years of age able to recite two related rules although they may still have difficulty displaying them in a conflict task [15, 16]. Success on simple working memory tasks such as the Piagetian A-not-B task is possible at 23 months and continues to improve throughout the preschool years [16]. However, cognitive flexibility is not well developed in preschool children and they may continue to exhibit rule perseverance even when tested again after a 1 month delay [17].

The importance of EF to a child’s developmental trajectory is underscored by the association between poor executive function and attention deficit disorder [18, 19], autism spectrum disorder [20, 21] and fetal alcohol spectrum disorder [22]. Perinatal events can result in developmental impairments [23], with the neural pathways controlling EFs particularly vulnerable [24]. Due to advances in medical interventions, increasing numbers of infants are born at risk of preterm birth, born small or born to diabetic mothers [25, 26], resulting in calls for further research on the neuropsychological outcomes of babies born at-risk [27, 28]. In practical terms, it behooves us to develop assessment processes for this growing number of preschoolers so that any delay in development can be identified and early intervention provided.

### The components of EF

EF is commonly divided into two conceptually different aspects involving different neural circuits; “hot” and “cold”, where hot EF tasks are emotionally charged, involving desire and avoidance, and cold tasks are rational cognitive tasks involving inhibition of thought or non-emotionally charged functions [29]. Until recently most assessment of childhood EF has focused on cold EF tasks, relying on modification of adult assessments. This has revealed that EF development begins around one year of age and continues into adulthood with the development of the prefrontal cortex [29]. Hot EF tasks are considered more difficult to manage in childhood because of the affective component. However, development of this aspect of EF appears to begin as early as 15 months and also continue into adulthood [30]. Further understanding of the relative importance of hot and cold EF comes from studies of Attention Deficit Hyperactivity Disorder where hot EFs are associated with hyperactive behaviors and cold EFs with inattention [31]. Further, in studies of the effect of maternal smoking during pregnancy, exposed children were found to be at risk of impairment of hot, but not cold, EF [32].

#### Attention and inhibition

Attention is the control of focus to selected perceptual input or cognitive processes. It appears in infancy and continues to develop throughout childhood [2], with attentional style at 5 months associated with EF in early childhood [33]. In early childhood, attention includes sustained and selective attention, and by 6 years attentional control can be identified [34]. Attention allows development of concentration, the screening out of distracting input and ignoring of prepotent responses [2, 10]. Attentional control is closely associated with inhibition, which includes both behavioral and cognitive control which are strongly correlated [35]. Inhibition underpins self-control and delayed gratification, with inhibition in early childhood positively associated with later outcomes in academic achievement, health, risk-taking, happiness and socioeconomic status [5, 10, 36].

#### Working memory

Working memory is the ability to manipulate and adjust information held in mind and has reciprocal relationships with both attentional and inhibitory processes [37, 38]. The theoretical underpinnings of working memory have been well described although overall agreement as to its structure is yet to be achieved [39, 40]. Working memory has an important association with mathematics, science, reading and overall educational success [7, 41, 42]. It has been shown to be closely aligned with both fluid and crystallised intelligence [10, 43].

#### Cognitive flexibility

Cognitive Flexibility, sometimes referred to as set-shifting, is the ability to change perspective and generate novel responses and is the last core EF to develop, [40, 44, 45]. It is an important aspect of goal management and creativity, develops rapidly in early childhood and is enhanced in young children by stimulating environments and resources and hence, is sensitive to family socio-economic status (SES) [46, 47]. Impaired cognitive flexibility is characteristic of Autistic Spectrum Disorder and is also associated with very preterm birth [21, 48]. It is important to note that the separate EFs are interdependent and in practice do not function in isolation. So whilst cognitive flexibility is the last component it does not occur without working memory, inhibition and focused and sustained attention [10] and can be thought of as an expressed combination of the previously developed EFs.

There is growing evidence of the efficacy of EF training for children with deficits [49–51] and of the importance of early intervention to improve life-course trajectories of at-risk infants [52, 53]. Two years is a common age for assessment of many at-risk cohorts to predict later development and neurosensory outcomes [54]. However, the lack of sensitive measures of cognitive development at this age has previously given rise to the description of this as the “dark ages” of cognitive development [55, 56]. Furthermore, the assessments typically undertaken at this age are developmental assessments that show only moderate correlation with later IQ [54, 57]. Given the association between EF and later development, the construction of an age-appropriate EF assessment battery for 2-year-olds would be valuable as it would allow early detection of difficulties in time for targeted early intervention. Simplification of EF assessment to allow a single measure of EF has practical advantages [58]. However, assessment of separate EFs at an early age has the potential to provide a useful diagnostic approach to understanding behavior if a preschooler is struggling to respond in an age-appropriate fashion. This approach, more than a summary score for EF or cognitive flexibility, can provide information on skill-specific interventions.

### Understanding EF in context

EF assessments should ideally be developmentally appropriate, assess individual components of EFs separately, and allow for a range of abilities without floor or ceiling effects. They should also have ecological and ethological (meaningful) validity [59]. However, it needs to be understood that task performance in quantitative assessment of EF is likely to depend on multiple cognitive factors. Examples of this would be the language skills required to understand or respond to verbal instructions and other EF skills such as the attention and inhibition required to prevent distractions and, say, allow updating of working memory. Increasingly EF assessments have employed tasks which focus on singular EF constructs derived from experimental psychology [9, 60] and age appropriate measures have allowed assessment of EF processes even within the first year after birth [10]. The interrelated nature of EF assessments and the reliance on language suggests that whilst simplified tasks may focus primarily on a single EF it is unlikely that assessment tasks can be developed for a single EF [2].

Neurodevelopmental outcome needs to be understood in the context of mediating factors such as family SES and the factors associated with this. There are many different measures of SES and it is uncertain which are the most appropriate measures for use in developmental studies, with logistical aspects of data collection, validity and comparability all issues that need to be considered [61, 62]. Poor EF outcomes are associated with social disadvantage in 2-year-olds where social disadvantage was a composite of education, employment, income, neighborhood and housing [56] and with low maternal education in 4-year-olds [63]. Low socioeconomic status has been associated with poorer infant and early childhood working memory [64, 65] and cognitive control in 10 to 13 year olds [66]. Preschool EF tasks contributing to the understanding of the association between SES and EF have previously been reported [67] but not for children as young as 2 years. We therefore developed a battery of EF assessment tasks, each designed to assess a single EF, which were quick, engaging and appropriate for use with 2-year-olds [9]. This battery was used to assess EF in a large group of toddlers born at risk of neonatal hypoglycemia and for whom we collected comprehensive SES data. The purpose of this report is to: (a) provide a description of this battery (b) report the results of its use in a clinical population and (c) report the impact of family SES on assessed EF at 2 years (d) provide suggestions as to how this battery may be further developed for use in preschool populations.

## Methods

### Sample

**Fig 1:**
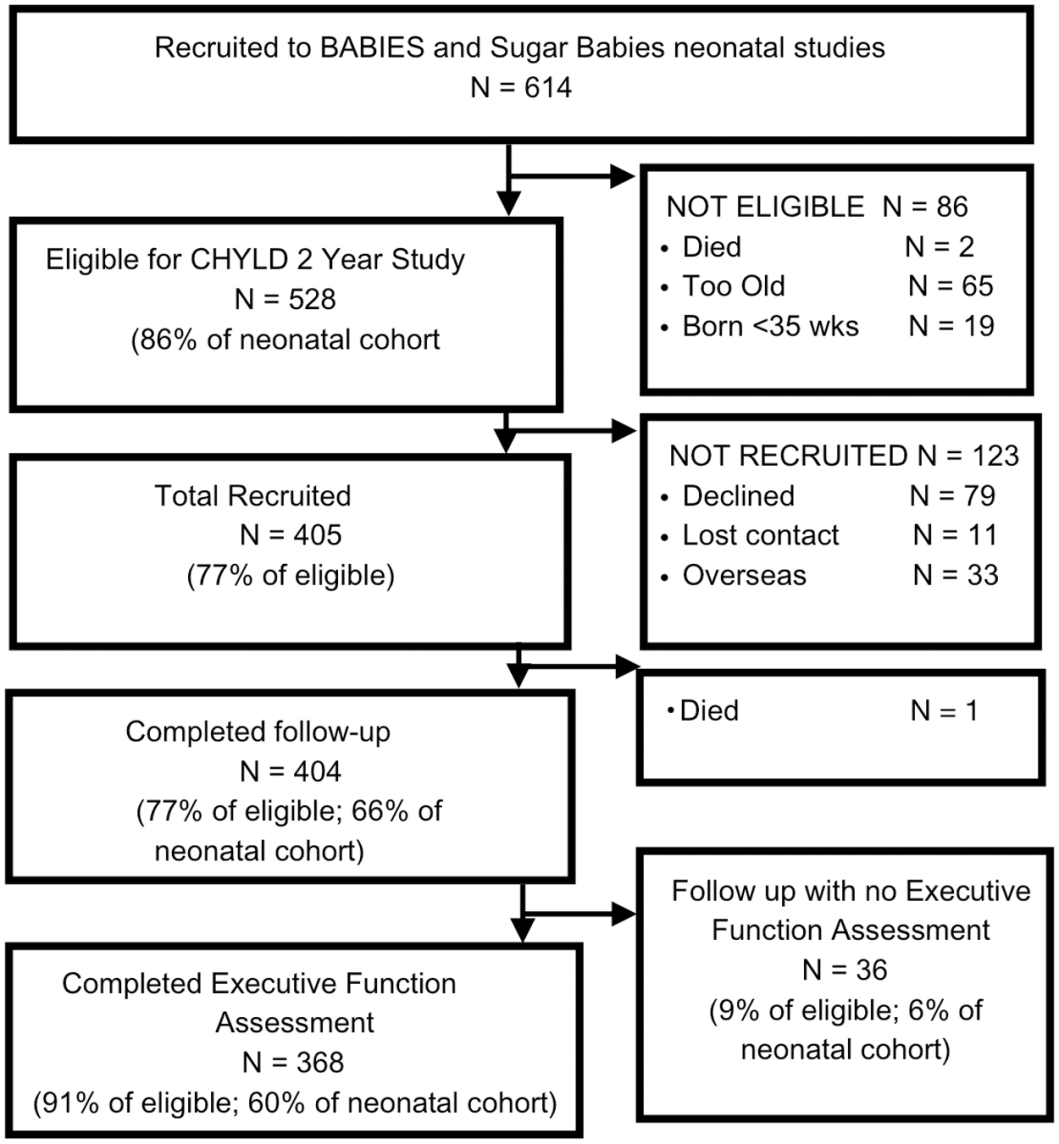
CHYLD study STROBE flow chart.

Children were born at the same New Zealand hospital between November 2008 and November 2010. Infants at birth were recruited due to their risk of neonatal hypoglycemia (infant of a diabetic mother (IDM), large (≥ 90^th^ percentile or ≥ 4500g), small (≤10^th^ percentile or ≤ 2500g), pre-term (35 - < 37 completed weeks gestation), other (sepsis, poor feeding) and had been recruited into a randomized trial of dextrose gel for treatment of neonatal hypoglycemia (Fig 1) [68].

### Procedure

Children attended for assessment with their primary caregiver at 24 ± 1 months at our research facility or a local clinic. Home assessments were conducted if no other options were practicable (n = 16). Assessments were conducted by one of five assessors, blinded to neonatal health status and trained to reliability, defined as being able to perform an assessment in accordance with the protocol, with a high degree of inter-rater reliability, as determined by video review by an experienced trainer (TAW).

The assessment took approximately 3 hours, including breaks as needed. It included the Bayley Scales of Infant and Toddler Development– 3^rd^ edition (Bayley III) [69], the EF battery that consisted of 4 EF tasks, an assessment of vision and global motion perception (30–45 minutes) and a pediatric examination (15–20 minutes). The order of assessment tasks varied for logistical reasons related to availability of the assessors. EF assessment occurred either in the first half of the assessment (First) after the Bayley III assessment, or at the end of the assessment (Last) following all other assessments.

Written consent was obtained from a parent or legal guardian prior to each assessment. Ethical approval was gained from Northern Y Health and Disability Ethics Committee (NTY/10/03/021).

### Measures

Each child’s primary care-giver completed questionnaires including home address, household income, level of educational attainment and health. Home address was used to obtain New Zealand Deprivation Index (NZDep) decile rating for each family. This measure uses census data to create small population group deprivation scores based on income, housing measures, employment and access to transport and communication. Nationwide, these scores are assigned to a decile rating with one indicating least and ten, most, deprived, so a low decile rating indicates high SES [70, 71]. Ratings are updated with each census and the iteration we used was based on 2006 data (NZDep2006).

#### Executive function tasks

These were based on previously reported assessments [72–75] modified for portability and increased toddler appeal (Table 1). The four tasks were offered in a standard order and a standardised script was used (S1 Appendix). Each began with a training task to familiarise the child with the task and to assess language competence. Failed trials were coded for the reason for failure, such as insufficient language or behavior problems, including refusal.

**Table 1:** Assessed EF tasks in order of presentation, showing domains assessed and scoring.

Task order	Task	EF Domain	Assessment Overview	Equipment	Maximum Score
1	Snack Delay (Kochanska et al., 2000)	Inhibition	Assesses child’s ability to inhibit a prepotent response. Here the prepotent response is to retrieve the treat, without waiting as instructed. *Success* = waiting for a minimum of 5 seconds and until the bell is rung before touching the treat.	Small mat, clear plastic cup, food treats, stopwatch, hand bell	8
2	Fruit Stroop (Kochanska et al., 2000)	Attention, inhibition	Assesses child’s ability to attend to the task of finding each little fruit and to inhibit their previous response, which was to point to each big fruit. *Success* = able to inhibit prepotent response and point to 1 or more little fruit.	Fruit Stroop cards: 1. Cards of big and little apple, orange, banana; 2. Cards of big fruit with different little fruit embedded	6
3	Ducks—Reverse Categorization (Carlson et al., 2004)	Working memory, cognitive flexibility	Assesses child’s ability to update a previously learnt sorting rule and actively maintain the new (reversed) rule. *Success* = reaching the criterion and correctly reverse sorting 3 or more ducks	Two children’s plastic buckets (1 big, 1 little); 3 little plastic ducks; 3 big plastic ducks	12
4	Multisearch Multilocation (Zelazo et al., 1998)	Working memory, cognitive flexibility	Assesses child’s ability to update a previously learnt location and maintain it for a short delay. *Success* = correctly locating treat on first attempt after location switch	Three drawer equipment; food treats; stopwatch	9
Total					35

#### Snack Delay

A treat was placed on the mat underneath an upturned cup and the child was encouraged to retrieve it. If the criterion of two successful training trials was met, the task instruction of waiting for the bell to be rung before retrieving the sweet was explained. This instruction was repeated between trials. The delay imposed increased with each trial (5, 15, 30 and 45 seconds’ delay).

#### Possible outcomes

1. Full wait: Waiting until the bell is rung before retrieving treat
2. Partial wait: Lifting or touching glass, but not treat, prior to bell being rung
3. Failed trial: Retrieving treat or ringing bell prior to bell being rung by assessor

The assessment continued until all four trials were completed or until the first failed trial. Two points were given for each full wait and 1 point for each partial wait, giving a maximum score of 8. If failure occurred on the first trial this was recorded as 0 seconds.

#### Fruit Stroop

The child was shown two series of pictures of an apple, orange and banana, one large and one small, and then asked to point to each large picture as it was named to check for language comprehension. Feedback was provided for both correct and incorrect responses. The child was then shown pictures of three small fruit (orange, apple and banana) each embedded in a picture of a different fruit (e.g. banana embedded in a picture of an orange) and asked to point to each of the named little fruit in turn, with no feedback given.

Two points were given for each correct response and 1 point if the child pointed to the big, rather than little, version of the fruit, giving a maximum of 6 points.

#### Reverse categorization (Ducks)

The child was taught to put the big toy duck in the big bucket and the little toy duck in the little bucket. Understanding of the rules was checked and feedback given.

The child was then shown an assortment of 3 large and 3 small ducks and asked to put each duck in the correct bucket. If the child correctly sorted at least 5 ducks, reverse categorization was introduced as a “silly game” in which the child was told that the big ducks were to be put into the little bucket and the little ducks should be put in the big bucket. Understanding of the new rules was checked and feedback given. The child was then shown an assortment of 3 big and 3 little ducks and asked to put the little duck in the big bucket and the big duck in the little bucket. One point was awarded for each duck correctly sorted in each part of the assessment, giving a maximum of 12 points.

#### Multisearch Multilocation

The treat was placed in the middle of three drawers, to which a black diamond shape was attached. The black felt cover was put over the drawers, the assessor told the child there was a treat, demonstrated lifting the black felt, and encouraged the child to open the drawer to retrieve the treat. The criterion to proceed to the pre-switch trials was that after three training trials the child was able to retrieve the treat without assistance.

Following training, the black diamond shape was removed and three different shapes were attached in a standard order: a yellow circle, blue triangle and green square. In the first set of trials (pre-switch) the food treat was always hidden in the middle ‘blue triangle’ drawer. The child watched the treat being ‘hidden’ and was encouraged to retrieve it.

A successful trial was recorded if the child found the treat on their first attempt. If the child opened an incorrect drawer the equipment was withdrawn and a failed trial was recorded. The correct drawer was then opened to reveal the treat and the instructions were repeated. Pre-switch trials continued until the child achieved three consecutive correct trials, (the criterion for progressing to the post-switch phase), or until six trials were attempted. Failure to respond after 30 seconds was a failed trial.

The post-switch phase was introduced as a “silly game” and the child was encouraged to watch as the food treat was hidden in the end ‘green square’ drawer. A 10 second delay was imposed before the child was presented with the drawers and encouraged to find the treat.

#### Possible outcomes

1. Post-switch success: retrieving the treat at the green square drawer
2. Perseverative error: unsuccessfully searching at the blue diamond
3. Non-perseverative error: unsuccessfully searching at the yellow circle

Trials continued until the child had correctly searched on two consecutive trials, or until eight trials had been attempted. Scores for pre-switch searching ranged from 1 to 3 (1 point for each correct response) and post-switch searching from 1 to 6 (reverse scored with 6 awarded for the first two searches both being correct, with score decreasing with number of attempts), giving a maximum score of 9.

#### EF total score

Scores on each assessed EF task were summed to give an EF total score, with a possible maximum of 35.

### Analysis

**Fig 2:**
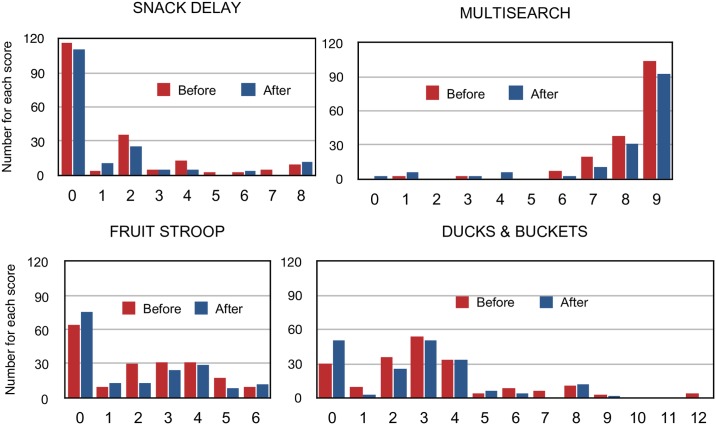
Distributions of those children who were tested at the beginning of the assessment and those children who were tested at the end of the assessment period. Higher scores are associated with better performance.

**Fig 3:**
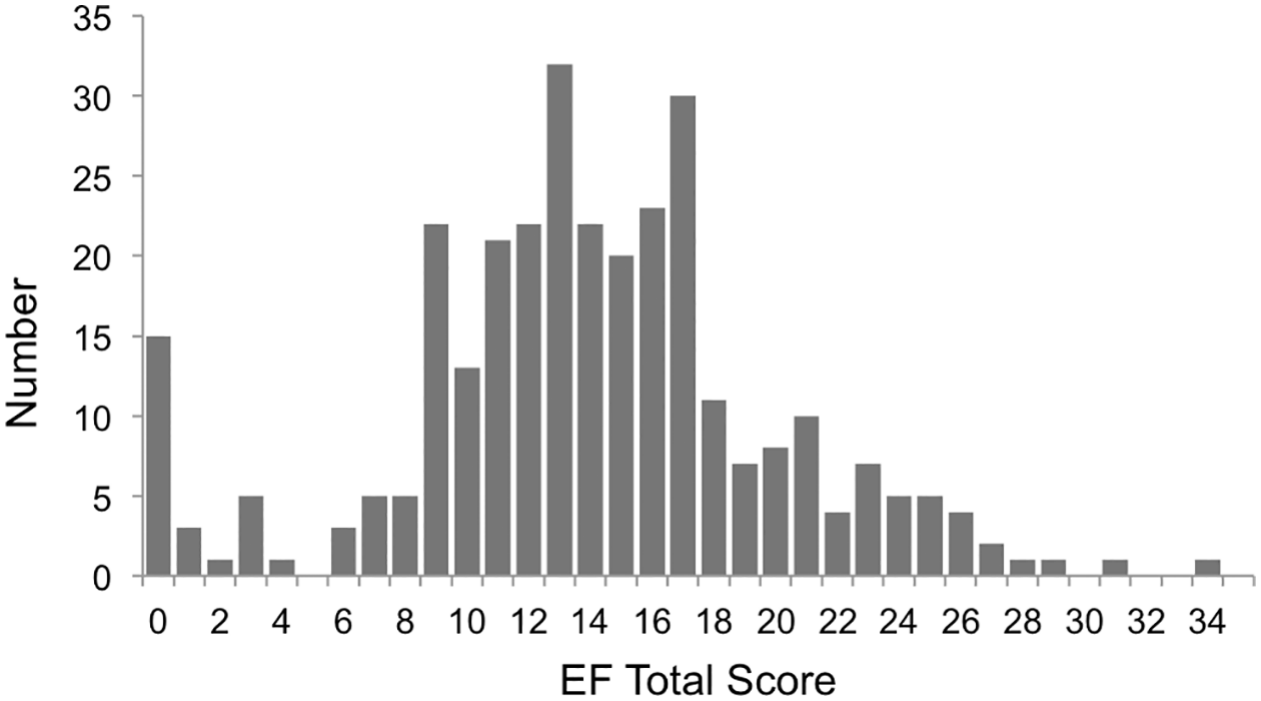
Distribution of the executive function total task score.

The Snack Delay, Fruit Stroop and Ducks assessments had right skewed distributions, and Multisearch Multilocation a left skewed distribution (Fig 2), which resulted in an approximately normal distribution for the EF Total score (Fig 3), with a small peak at 0. Results are reported as mean (SD) rather than median (95% CI) to allow for easier comparison with other reports.

To determine the proportion of children who successfully completed each level of the individual tasks, children were allocated to groups based on the highest level they achieved on each task. For Snack Delay, the four groups were 0 seconds (failure), 5, 15, ≥ 30seconds. For Fruit Stroop, the three groups were the total number of correct small fruit identifications made. For Ducks the four groups were < 5 ducks correctly sorted, 5–6 ducks correctly sorted, 1–2 ducks correctly reverse sorted, and ≥3 ducks correctly reverse sorted. For Multisearch Multilocation the two groups were pre-switch success and post-switch success. Chi-square analyses were used to compare groups and results are presented as success rates (n, % of cohort). Task success was defined as performing the minimum required to demonstrate the EF (Table 1). Continuous data (task scores) were compared between groups using Independent samples Mann-Whitney U for dichotomous variables; and Kruskal-Wallis (1-way ANOVA, k samples) for multiple variables using pairwise comparisons with Bonferonni corrections for multiple comparisons.

Data pertaining to SES were recoded to reduce the number of categories and create a more even spread between categories. Household income was grouped as: High ≥ $70,000, Medium = $70,000 - $40,000, Low ≤ $40,000. NZDep2006 deciles were grouped as: High: Deciles 1–3, Medium: Deciles 4–6, Low: Deciles 7–10. Parent reported highest education level was grouped as: High: University education, Medium: Post-secondary training such as trade certificate or Polytechnic, Low: Secondary education or less.

## Results

Maternal and infant characteristics of the sample are provided in Table 2. From the neonatal cohort of 528 children, 404 (77%) were recruited to this follow-up study. Of these, EF data were available for 368 (91.1%) of those followed up, of whom 193 (52.4%) were boys. Within this group, 193 (52.4%) had experienced neonatal hypoglycemia (at least one blood glucose concentration <2.6 mmol/L). No neonatal seizures were recorded. The mean (SD) gestational age was 37.7 (1.6) weeks and birth weight 3123 (840) grams. The most common risk factor was infant of a diabetic mother (147 children, 40.0%) with 124 (33.7%) late preterm, 50 (13.6%) small, 36 (9.8%) large and 11 (3%) other. Infant ethnicity identified by the primary caregiver were: New Zealand European 181 (50.8%), Maori 132 (37.1%), Pacific Islands 18 (5.1%), Asian and other 25 (7.0%). There was a higher proportion of children from low than from medium or high SES families, with 64 (16.8%) in the high group, 120 (34%) in the medium group and 183 (50.3%) in the low group. Household income data were available for 304 (82.6%), NZDep2006 for 364 (98.9%) and maternal education for 352 (95.7%).

**Table 2:** Maternal and infant characteristics of participants.

Maternal & Infant characteristics	N	N (%) or Mean (SD)
**Maternal characteristics**		
Maternal Age	368	29.9 (6.3)
Maternal Education	344	
*High school/secondary school or less (low)*		111 (32.3)
*Post high school training (medium)*		109 (31.7)
*University education (high)*		124 (33.7)
Socioeconomic status		
Deprivation Index	364	
*Deciles 1–3 (High SES)*		61 (16.8)
*Deciles 4–6 (Medium SES)*		120 (33.0)
*Deciles 7–10 (Low SES)*		183 (50.3)
Yearly household income	299	
*<$40*,*000*		112 (37.5)
*$40*,*001 - $70*,*000*		86 (23.4)
*>$70*,*001*		101 (27.4)
Maternal substance use during pregnancy	359	
*Tobacco*		96 (26.1)
*Alcohol*		32 (8.7)
*Marijuana*		38 (10.3)
**Infant Characteristics**		
Male	368	193 (52.4)
Gestation (wk)		37.72 (1.63)
Primary risk factor		
*Infant of diabetic mother*		147 (39.9)
*Preterm*: *<37 completed weeks*		124 (33.7)
*Small*: *<10*^*th*^ *percentile or <2500 gms*		50 (13.6)
*Large*: *>90*^*th*^ *percentile or 4500 gms*		36 (9.8)
*Other*		11 (3.0)
Neonatal hypoglycaemia		193 (52.4)
Hospitalised for illness (birth-24 months)	346	108 (29.3)
Attending day care	311	160 (43.5)
EF examined first		192 (52.2)

N = 368 unless otherwise specified

### Infant characteristics and EF

EF scores did not differ between those children who had neonatal hypoglycemia and those who did not (McKinlay et al., 2015). There were also no differences between those infants born to diabetic mothers compared to those who were not, or for any of the other risk factors (born small, large or preterm). No differences were found between children who attended day care compared to those who did not. A significantly lower Total Task score was found between children who were hospitalized and those who were not during the period from birth to the 2-year assessment (p = 0.017). Significantly different scores were also found for ethnicity with children identified as New Zealand European performing significantly better than Maori (p = 0.008), Pacific Islands (p = 0.048) and Asian and Other (p = 0.016).

### EF and behavior

Data on the order of assessment were available for 366 (99.5%) children. Similar numbers of children were tested first (N = 192) and last (N = 174). When the EF assessment was conducted last, approximately three times as many children (11, 6.3% vs 3, 1.6% for those tested first) were excluded for behavioral reasons from the Snack Delay (p = 0.018) and Multisearch Multilocation tasks (p = 0.008), but there was no relationship between timing of assessment and behavioral exclusions from the Fruit Stroop or Ducks tasks (Table 3). Children whose EF assessment was conducted last had lower scores on the Ducks task (p = 0.004) and EF Total score (p = 0.013), but similar scores for all other tasks.

**Table 3:** Behavioral failures and EF task scores for children whose EF assessment was conducted in the first half of the assessment or last.

		Failed for behavior		Task Score	
		First	Last	First	Last
	Cohort n	192	174		
	Girls n	87	86		
	Boys n	105	88		
**Snack Delay**					
	Cohort	3 (1.6)	11 (6.3)*	1.4 (2.2)	1.3 (2.3)
	Girls	2 (2.3)	5 (5.8)	1.7 (2.4)	1.4 (2.3)
	Boys	1 (1.0)	6 (6.8)	1.2 (2.1)	1.3 (2.3)
**Fruit Stroop**					
	Cohort	37 (19.3)	44 (25.3)	2.2 (1.9)	1.9 (2.0)
	Girls	15 (17.2)	24 (27.9)	2.4 (1.9)	1.8 (1.9)
	Boys	22 (21.0)	20 (22.7)	2.0(1.9)	2.1 (2.1)
**Ducks**					
	Cohort	33 (17.2)	39 (22.4)	3.3 (2.6)	2.5(2.1)***
	Girls	10 (11.5)	16 (18.6)	3.7 (2.6)	2.8(2.0)
	Boys	23 (21.9)	23 (26.1)	3.0 (2.5)	2.3 (2.2)
**MSML**					
	Cohort	6 (3.4)	16(11.0)**	8.4 (1.1)	8.4 (1.3)
	Girls	4 (4.7)	7 (9.5)	8.3 (1.4)	8.6 (0.9) ^i^
	Boys	2 (2.2)	9 (12.5)	8.5 (0.8)	8.0 (2.1)
**EF Total**					
	Cohort			14.7 (5.6)	13.0 (6.6)*
	Girls			15.1 (6.0)	13.8 (6.0)
	Boys			14.3 (5.3)	12.2 (7.1)

Data are N (%) who failed the task for behavior and mean (SD) for Task score;

*p<0.05, **p<0.01, *** p<.001 for comparison with first assessment;

^**i**^ p<0.05 for interaction between sex and assessment order;

MSML = Multisearch Multilocation

There was a significant interaction between sex and assessment order for Multisearch Multilocation, with boys, but not girls, whose assessment was conducted last having lower scores (p = 0.014).

The EF assessment took approximately 15 minutes and children engaged readily and appeared to enjoy the process.

#### Snack Delay

Nearly two-thirds of children (228, 62.0%) were unable to inhibit their prepotent response and failed the first test condition (0 second), with 12 of these for behavioral reasons (Table 3). Success decreased with increased delay. Girls had a higher overall success rate than boys (p = 0.027), although there was no significant difference in mean Snack Delay score between girls and boys.

#### Fruit Stroop

More than half of children (206, 56.0%) failed to inhibit their prepotent response and attend sufficiently to identify any of the small fruit, with 76 (20.7%) children failing for behavioral reasons (Table 3). The little apple was the fruit most frequently correctly identified (80 (21.7%) children). There were no differences between girls and boys for success rates or Fruit Stroop score.

#### Ducks/Reverse categorization

Most children (315, 85.6%) correctly sorted some ducks although few (53, 14.4%) reached the criterion for the reverse categorization task by correctly sorting five or six ducks (Table 3). Of those who attempted reverse categorization, 20 (37.7%; or 5.4% of total cohort) correctly reverse sorted 1 or 2 ducks and 11 (20.8%; or 3.0% of total cohort) 3 or more. At the categorization task stage 61 (16.6%) and at the reverse task stage 7 (1.9%) children failed for behavioral reasons. Success rates were similar in girls and boys, although girls had a higher Ducks score than boys (p = 0.022).

### Multisearch Multilocation

Most children (312, 84.8%) were able to complete the pre-switch task, which was the criterion for progressing to the Post-switch task (Table 3), although 23 (6.3%) children failed for behavioral reasons. More than half (216, 58.7%) of the cohort achieved success on the first Post-switch trial. Of those who failed at this stage, 108 (29.3%) made a perseverative error and 2 (0.6%) a non-perseverative error. There were no significant differences between girls and boys in Multisearch Multilocation success or score.

### EF total score

Fifteen children (4.1%) had a total score of 0. The EF total score did not differ significantly between girls and boys.

### Success rates

The four tasks had a range of success rates (Table 4). Multisearch Multilocation had the highest success rate (58.7%) and Ducks/Reverse Categorization the lowest (3%) with similar rates for Snack Delay (38%) and Fruit Stroop (44%).

**Table 4:** Success rates and total scores for executive function tasks for total cohort, girls and boys.

Assessment	Total N = 368	Girls N = 175	Boys N = 193
**Snack Delay**			
0s failure	228 (62.0)	99 (56.6)^†^	129 (66.8)
5s success	72 (19.6)	44 (25.1)	28 (14.5)
15s success	30 (8.2)	11 (6.3)	19 (9.8)
≥ 30s success	38 (10.3)	21 (12.0)	17 (8.8)
*Snack Delay Score*	1.4 (2.3)	1.5 (2.3)	1.2 (2.2)
Task success	140 (38.0)		
**Fruit Stroop**			
0 correct	206 (56.0)	101 (57.7)	105 (54.4)
1 correct	104 (28.3)	47 (26.9)	57 (29.5)
2 correct	37 (10.1)	18 (10.3)	19 (9.8)
3 correct	21 (5.7)	9 (5.1)	12 (6.2)
*Fruit Stroop score*	2.1 (2.0)	2.1 (1.9)	2.1 (2.0)
Task success	162 (44.0)		
**Ducks**			
< 5 categorized	315 (85.6)	147 (84.0)	168 (87.1)
5–6 categorized	22 (6.0)	10 (5.7)	12 (6.2)
1–2 reverse	20 (5.4)	13 (7.4)	7 (3.6)
≥ 3 reverse	11 (3.0)	5 (2.9)	6 (3.1)
*Ducks score*	2.9 (2.4)	3.2 (2.4)^††^	2.7 (2.4)
Task success	11 (3.0)		
**MSML**			
Pre-switch success	312 (84.8)	151 (86.3)	161 (83.4)
Post-switch success	216 (58.7)	109 (62.3)	107 (55.4)
*MSML score*	8.4 (1.2)	8.4 (1.2)	8.4 (1.2)
Task success	216 (58.7)		
**EF Total Score**	14.0 (6.1)	14.5 (6.0)	13.4 (6.2)

Data are n (%) or mean (SD).

^**†**^p<0.05 and ^**††**^p<.01 for difference between girls and boys.

MSML = Multisearch Multilocation. See text for definitions of task success.

#### EF and socioeconomic status

Children from low income households had lower scores on all four EF tasks and lower EF Total score than those from high income households. They also had significantly lower scores than children from medium income households on Fruit Stroop, Snack Delay and the Total EF Score (Table 5).

**Table 5:** Executive function scores for the cohort and girls and boys for high, medium and low groups of household income, neighbourhood deprivation and maternal education.

		Household income at 2 year assessment			NZDep2006 at 2 year assessment			Maternal Education		
		High	Medium	Low	High	Medium	Low	High	Medium	Low
	Cohort N	101	86	112	61	120	183	112	113	126
	Girls N	51	49	49	27	64	83	52	59	55
	Boys N	50	38	66	34	56	100	60	54	71
**Snack Delay**	Cohort	1.9	1.8	1.0 ^a^^bb^	1.8	1.8	1.0 ^bb^	1.7	1.3	1.2
		(2.7)	(2.3)	(1.9)	(2.7)	(2.5)	(1.9)	(2.5)	(2.1)	(2.1)
	Girls	1.9	2.2^†^	0.9 ^a^	2.3	1.7	1.1	2.2	1.3	1.4
		(2.7)	(2.5)	(1.8)	(3.0)	(2.6)	(1.8)	(2.7)	(2.2)	(2.2)
	Boys	1.8	1.2	1.0	1.4	1.9	0.9 ^bb^	1.3	1.2	1.1
		(2.7)	(1.9)	(2.0)	(2.5)	(2.3)	(2.0)	(2.2)	(2.1)	(2.0)
**Fruit Stroop**	Cohort	2.6	2.4	1.7 ^aa^^bb^	2.5	2.3	1.8	2.2	2.3	1.8
		(2.0)	(1.9)	(2.0)	(2.1)	(2.0)	(1.9)	(2.0)	(2.0)	(2.0)
	Girls	2.5	2.6	1.5 ^a^^b^	2.8	2.3	1.7 ^a^	2.3	2.3	1.9
		(2.0)	(1.9)	(1.8)	(2.1)	(1.9)	(1.9)	(1.9)	(2.0)	(2.0)
	Boys	2.7	2.2	1.8	2.2	2.3	1.9	2.1	2.4	1.8
		(2.0)	(1.9)	(2.1)	(2.1)	(2.1)	(1.9)	(2.1)	(1.9)	(2.0)
**Ducks**	Cohort	3.9	3.1	2.4 ^aaa^	3.2	3.3	2.7	3.4	3.2	2.5 ^a^^bb^
		(2.8)	(2.3)	(2.0)	(2.6)	(2.3)	(2.4)	(2.7)	(2.5)	(2.0)
	Girls	4.1	3.4	2.6 ^aa^	3.6	3.5	3.0	3.8	3.6	2.7 ^a^
		(2.5)	(2.5)	(2.0)	(2.3)	(2.2)	(2.5)	(2.3)	(2.6)	(2.1)
	Boys	3.6	2.6	2.3 ^a^	2.9	3.0	2.5	3.0	2.8	2.3
		(3.2)	(2.1)	(2.0)	(2.8)	(2.4)	(2.3)	(3.0)	(2.3)	(1.9)
**MSML**	Cohort	8.4	8.6	7.9 ^bbb^	8.3	8.6	8.2^b^	8.4	8.5	8.2
		(1.0)	(0.9)	(2.1)	(1.7)	(0.9)	(1.5)	(1.3)	(0.9)	(1.6)
	Girls	8.5	8.6	8.0	8.2	8.7	8.3	8.5	8.6	8.4
		(0.9)	(1.1)	(1.8)	(2.2)	(0.9)^†^	(1.0)	(1.1)	(0.7)	(1.2)
	Boys	8.3	8.7	7.7 ^b^	8.3	8.4	8.1	8.3	8.6	8.2
		(1.1)	(0.6)	(2.2)	(1.5)	(1.2)	(1.8)	(1.3)	(0.7)	(1.8)
**EF Total**	Cohort	15.6	15.0	11.7^aaa^^bbb^	14.5	15.2	12.5^bbb^	14.6	14.2	12.5^a^
		(6.2)	(6.0)	(5.9)	(7.1)	(5.4)	(5.9)	(6.4)	(5.8)	(5.9)
	Girls	15.6	16.3	11.8^aa^^bbb^	15.1	15.6	13.0 ^b^	15.9	14.6	13.0
		(6.4)	(5.9)^†^	(4.9)	(7.5)	(5.2)	(5.7)	(5.7)	(6.2)	(6.1)
	Boys	15.7	13.3	11.6 ^aa^	13.9	14.7	12.1 ^b^	13.4	13.7	12.1
		(6.1)	(5.6)	(6.5)	(6.8)	(5.5)	(6.0)	(6.8)	(5.4)	(5.8)

Data are Mean (SD);

^†^p<0.05; ^††^p<0.01 for comparison between sexes;

^a^p<0.05; ^aa^p<0.01; ^aaa^p<0.001 for comparison with High groups;

^b^p<0.05; ^bb^p<0.01; ^bbb^p<0.001 for comparison with Medium and Low groups;

Household income: High = ≥ $70,001/year; Medium = $40,000 - $70,000/year; Low = ≤ $40,000/year;

NZDep2006; High = deciles 1, 2, 3; Medium = deciles 4, 5, 6; Low = deciles 7, 8, 9, 10;

Maternal Education: High = University education; Medium = Post-secondary training such as Polytechnic or trade certificate; Low = Up to completion of secondary (high) school.

Children from families in the low SES group, as measured by NZDep2006, had lower Snack Delay Scores, MSML scores and EF Total scores than those from families in the medium NZDep2006 group.

A low level of maternal education was also associated with lower EF scores, with the low maternal education group having significantly lower scores on Ducks and EF Total.

There were no significant interactions between sex and household income, NZDep2006 at birth or 2 years or parent education for any of the scores.

### Relationships between EF measures

The Snack Delay, Fruit Stroop and Ducks scores were each significantly correlated with the other task scores, but only accounted for a small amount of their variation (4–8%). Scores on the Multisearch Multilocation task were not correlated with scores on any of the other tasks (Table 6), and nor were scores on the Post-switch task. Cronbach’s alpha for all four tasks was 0.429, and omitting the Multisearch Multilocation task was 0.491.

**Table 6:** Relationships between assessed executive function task scores.

	**Snack Delay**	**Fruit Stroop**	**Ducks**	**Multisearch Multilocation**
**Snack Delay**		R^2^ = 0.040	R^2^ = 0.077	R^2^ = 0.003
	-	β = 0.229***	β = 0.262***	β = 0.103
		[0.113, 0.344]	[0.169, 0.355]	[-0.094, 0.299]
**Fruit Stroop**		-	R^2^ = 0.064	R^2^ = 0.0001
			β = 0.209***	β = 0.017
			[0.127, 0.291]	[-0.151, 0.185]
**Ducks**				R^2^ = 0.004
			-	β = 0.111
				[-0.095, 0.317]
**Multisearch Multilocation**				-

Data are R^2^, β [95%CI];

***p<0.001

**Fig 4:**
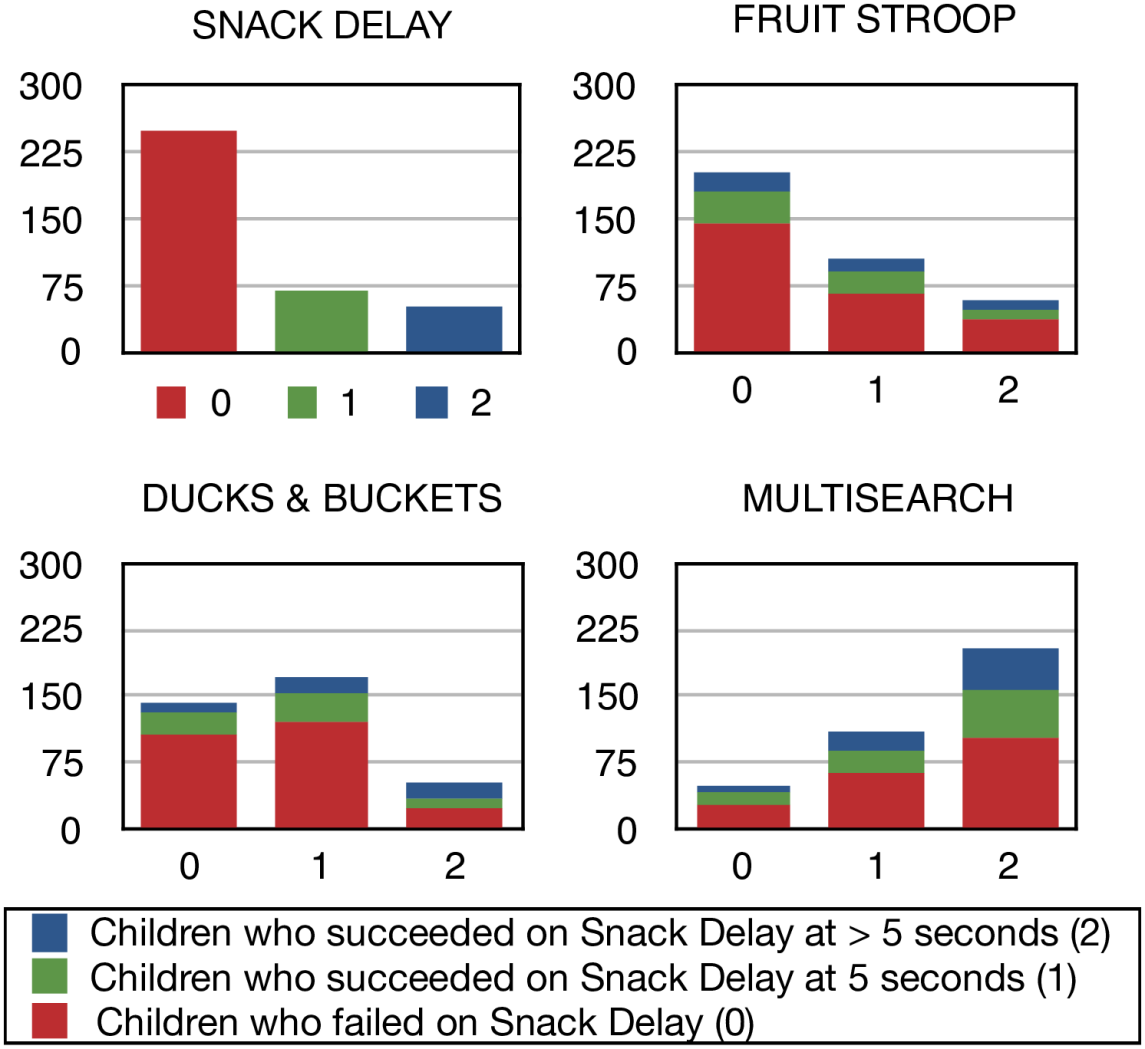
Distribution of results in all EF tasks showing the number of children who failed, succeeded partially and succeeded fully in the Snack Delay task.

Scores of individual children showed little consistency across tasks, as illustrated by children on each level of Snack Delay being represented at all levels of success in the other three tasks (Fig 4). However, approximately half of the children who achieved level 2 success on Snack Delay, indicating a good level of inhibitory control, also achieved level 2 success on Ducks, and very few of them were represented at level 0 for Multisearch Multilocation.

## Discussion

At the beginning of our study there were few reports of EF assessments for 2-year-olds. We used these early reports as a guide to assemble a developmentally appropriate and practical EF assessment battery [9, 72, 74, 75]. This was intended to include the principle components of preschool EF: attention, inhibition, working memory and cognitive flexibility; and to provide a range of difficulties and distribution of scores within each task while avoiding floor and ceiling effects. The success rates for the four tasks replicated the order of EF task difficulty previously reported by Carlson for a group of 118 2-year-olds (2005), providing confidence in the reliability of this assessment at this age. Furthermore, the differences described in response to differences in SES follow a pattern that would be expected based on results for older children [66, 76]. This large study demonstrates that it is possible to directly assess EF in 2-year-olds and provides us with sufficient data to suggest amendments that would allow a wider use of this assessment battery.

Emerging EF and other developmental domains including language and behavior are inter-related, highlighting the difficulties of developing preschool EF measures with a single focus [77]. The Ducks task, with the most complicated set of verbal instructions targets working memory, which is limited at this stage of development. Therefore, the lower scores on this task may be explained by differing levels of language ability in this age group or aspects of working memory that include the ability to attend to instructions and hold them in short term memory. Further development of this assessment could include simplifying the language and incorporating more modelling of behavior. The Ducks and Fruit Stroop tasks both required children to know the words ‘big’ and ‘little’, and to have developed the concept of comparative size. The Ducks categorization could be replaced by two groups of different toys, thereby removing the size knowledge component. The importance of language development for the Fruit Stroop task is also illustrated by the fact that while 28% of children correctly identified one little fruit, most of these (22%) identified the apple, the most commonly available fruit in New Zealand. Behavioral requirements were also important, and included attending sufficiently to the pictures to focus on a subdominant visual feature, inhibiting a response to the dominant visual feature, and responding as requested [74].

A four-step Multisearch Multilocation task had been reported [75], but our pilot study indicated that the motor demands were too great for many 2-year-olds, and we therefore used a three-step process. However, scores for this task may be more normally distributed if it provided greater challenge such as by increasing the number of drawers, providing a second switch phase or increasing the delay period [75].

Scores for the Snack Delay, Fruit Stroop and Ducks tasks were correlated with each other, although this accounted for only a small amount of the variation in each score and the Cronbach’s alpha was low. Furthermore, success on a single task did not predict success in other tasks. These findings suggest that the skills required were different for each task, and thus that the tasks were measuring distinct skills, potentially allowing identification of separate EF problems. Our results are consistent with the view that the functions which compose EF are separate but interrelated at 2 years and thus likely served by separate neural pathways which differentiate with increasing age and cognitive development [2, 60].

The data we collected on the timing of each assessment and children’s affective responses allowed us to understand a range of assessment factors that contribute to outcome. Children who had their EF assessment last in the assessment session were more likely to fail the Snack Delay and Multisearch Multilocation tasks for behavioral reasons, and also had lower scores for Ducks, leading to lower EF Total scores than children whose EF assessment was in the first half of the session. Behavior late in the session may have been influenced by tiredness and hunger, especially since both Snack Delay and Multisearch Multilocation are ‘Hot’ or emotionally charged rather than ‘Cool’ or abstract tasks [78]. Fruit Stroop and Ducks had the highest behavioral fail rates but these were not higher in children whose EF assessment was last, although scores for the Ducks task were lower for those children. This suggests that even for children who were able to manage their behavior, the task became more demanding when late in the assessment process. Our findings suggest that to achieve optimal assessment of 2-year-olds, practical considerations such as language used, length of assessment, scheduling within the assessment battery, and timing in relation to sleep times need to be included in planning. Ideally, EF assessment would be conducted in a single separate session without other scheduled assessments.

Although, 14 children could not complete the EF battery (3 in the group tested first and 11 in the group tested last), the majority of the children engaged readily with the EF assessment tasks, regardless of assessment order. Girls were more successful than boys in completing the Snack Delay and Ducks tasks, indicating a greater ability to inhibit a prepotent response and successfully employ working memory. This sex difference in inhibitory control and EF skills has been previously reported for preschoolers [79]. However, we also found that boys’, but not girls’, scores on the Multisearch Multilocation assessment of working memory were affected by assessment order, suggesting that tiredness or test fatigue was more likely to influence performance for boys. Nevertheless, there were no differences between boys and girls in the overall EF score, suggesting that this battery of tasks is appropriate for both sexes at this age [9], although may require separate standardization for girls and boys.

Our results also indicate that assessed EF, with narrow focus on target behaviors, can identify both children with EF deficits such as lack of inhibitory control or poor working memory, and those who have a high level of EF skills such as very good inhibitory control. We also identified a small group of children who scored no points on any of the four tasks. Overall, success rates appear to be lower for all tasks than those previously reported by Carlson (2005) as EF Task Difficulty. However, the cohort reported by Carlson was primarily “white and middle-class” (p 598), unlike our cohort born at neonatal risk with a high proportion of low SES families, further supporting the potential clinical usefulness of this battery of tasks in an at-risk group. The increasing numbers of children surviving neonatal risk factors such as preterm birth and diabetic pregnancies suggests that these data may provide a comparator group for future follow-up studies of at-risk newborns. They may also provide useful comparisons for cohorts of 2 year olds from families of mixed and low SES.

Our data show that household income is a significant SES factor associated with EF development in New Zealand children at 2 years, being more strongly and consistently associated with EF scores than maternal education or neighborhood deprivation. This is an important distinction as these variables are often used interchangeably in child development research. Elsewhere, association between EF and social disadvantage, including neighbourhood descriptors, has been found in children at 2 to 3 years of age [56] and between EF and maternal education at early school age [80, 81], with reports differing as to whether household income or maternal education is a stronger predictor of EF [62, 76]. Participant unwillingness to report family income means a comparison between these measures can be difficult [62]. However, we achieved an 82.6% reporting rate for household income and, although lower than the maternal education reporting rate (95.7%), this provided us with a good level of confidence in our analysis of SES. Elsewhere, neonatal risk and family SES were found to be separate but multiplicative in their effect on developmental delay [82] suggesting that an understanding of both is important to the understanding of developmental outcome in at-risk children.

Low SES has been associated with reduced success on the A-not-B task at 6–14 month olds [65], impaired set-shifting and attention deficit hyperactivity disorder (ADHD) in 3–6 year olds [83], and reduced problem-solving in 4-year-olds [82]. The three measures of SES we assessed (household income, maternal education and New Zealand Deprivation Index) all revealed poorer EF performance on some tasks for children from low SES families. However, the association between SES and EF task score differed with different measures. Whereas all task scores decreased with family income, only Snack Delay and EF Total score showed differences associated with NZDep2006 and only Ducks and EF Total score showed differences associated with maternal education. At school age childhood poverty has been found to have a greater effect on the development of neurocognitive systems underlying language, cognitive control and working memory than other systems [66, 84]. Our findings are in agreement with Hackman and Farah’s (2009) contention that different aspects of SES affect development in different ways, and support the notion that a complete understanding of the association between SES and development requires assessment of multiple measures [85].

The data reported here indicate that the EF assessment tasks we used are sensitive to socioeconomic factors, thereby providing support for their validity as age-appropriate assessments. Ideally, these tasks would be standardized with a larger, representative and healthy population of New Zealand children, as this would allow comparison of results between at-risk cohorts such as ours and expected New Zealand preschool performance. International standardization would provide an even greater usefulness in clinical and preschool settings. Further, it would be ideal to determine the extent to which the skills assessed at 2 years predict EF skills at a later age, and whether the separate scores or an EF composite measure are more predictive of later EF and behavioral outcomes. Continued follow-up of this cohort will allow us to answer these questions.

The main limitation of this study was a lack of a normative group of children for comparison. Furthermore, some assessments had to be carried out in the home, where uncontrollable distractions for the child may potentially have affected the scores in this group. However, the child tested in the home, a familiar environment, may also perform better for this reason, and there were no systematic differences in scores between those assessed in our lab and those assessed in the home.

There were also a number of strengths of this study, including the large sample size, narrow age range and rigorously administered and evaluated assessments [86]. Importantly, the details of method have been accurately reported to allow others the opportunity to further develop robust processes for EF assessment in the toddler years.

This current study contributes new information for early childhood educators and researchers by presenting results from a large cohort of 2-year-olds and demonstrates the feasibility of direct measurement of EF, even at this young age. We have presented our professional reflections on these assessments in anticipation that the modifications suggested will further enhance the specificity of the assessment tasks. The increasing numbers of babies surviving complicated pregnancies [25–27] (Blencowe et al., 2012; Gerner & Baron, 2014; Green et al., 2004) suggests attention needs to be paid to processes that may ameliorate the risk of long-term poor neuropsychological, and hence educational, outcome in these children. Others have reported that EF assessment tasks can successfully be conducted, with the results usefully complementing parent report, for 3–5 year olds born either preterm or full-term [87]. However, the assessment tasks described here have the potential to identify EF skills in children as young as 2 years. They appear to measure distinct aspects of EF, independent of sex, and are only modestly affected by language and behavioral issues. Importantly we showed that these EF measures were sensitive to different measures of SES that are often used interchangeably in child development studies. The modified battery of tasks presented here has the potential to contribute much-needed knowledge of EF development in typically and atypically developing children, a challenge thrown down by Carlson a decade ago [9, 29]. Early identification of EF impairment as a result of health or socioeconomic risk using EF assessments may allow appropriate early childhood intervention for children in high risk groups who may otherwise be at a developmental disadvantage [51, 52, 88]. The importance of EF in early childhood development to later academic and behavioral outcomes underscores the need for an appropriate battery of direct assessment tasks such as described here.

## Supporting information

S1 Appendix Executive function assessment script and scoring schedule. (DOCX)
