# Supplementary material for: Executive function assessment in New Zealand 2-year olds born at risk of neonatal hypoglycemia
Source: PLoS One. 2017 Nov 22;12(11):e0188158. doi: 10.1371/journal.pone.0188158 (PMC5699811; doi:10.1371/journal.pone.0188158)
Supplement: S1 Appendix — (DOCX) [file pone.0188158.s001.docx]

S1 Appendix: EF assessment script and scoring schedule

| **Study ID:** | **Birth Date:** |
| --- | --- |
| **Examiner’s No.** | **Date of Assessment:** |

CHYLD STUDY

Executive Function Summary Score Sheet

| **Snack Delay** | **Trial Error** | 🞏 (97) Examiner 🞏 (98) Child B 🞏 (99) Child L | | | | | | | |
| --- | --- | --- | --- | --- | --- | --- | --- | --- | --- |
|  | **Trial** | **Completed/Incompleted** | | | **Time to Treat** | | | | Retrieved |
|  | Practice 1 |  | | |  | | | | 🞏 N 🞏 Y |
|  | Practice 2 |  | | |  | | | | 🞏 N 🞏 Y |
|  | 0 | No Trials Completed | | | < 5 Seconds | | | | 🞏 N 🞏 Y |
|  | 5 seconds | **Full** 🞏 Yes 🞏 No | **Part** 🞏 Yes 🞏 No | | RT Seconds | | | | 🞏 N 🞏 Y |
|  | 15 seconds | **Full** 🞏 Yes 🞏 No | **Part** 🞏 Yes 🞏 No | | RT Seconds | | | | 🞏 N 🞏 Y |
|  | 30 seconds | **Full** 🞏 Yes 🞏 No | **Part** 🞏 Yes 🞏 No | | RT Seconds | | | | 🞏 N 🞏 Y |
|  | 45 seconds | **Full** 🞏 Yes 🞏 No | **Part** 🞏 Yes 🞏 No | | RT Seconds | | | | 🞏 N 🞏 Y |
|  | | | | | | | | | |
| **Fruit Stroop Task** | **Trial Error** | 🞏 (97) Examiner 🞏 (98) Child B 🞏 (99) Child L | | | | | | | |
|  | **Trial** | **Correct/Incorrect** | | | | | | | |
|  | Identifies | Apple | | 🞏 (1) Correct | | | 🞏 (0) Incorrect | | |
|  | Identifies | Orange | | 🞏 (1) Correct | | | 🞏 (0) Incorrect | | |
|  | Identifies | Banana | | 🞏 (1) Correct | | | 🞏 (0) Incorrect | | |
|  | Little Apple | 🞏 (1) Little Apple | | 🞏 (0) Big Apple | | | 🞏 (0) Other | | |
|  | Little Banana | 🞏 (1) Little Banana | | 🞏 (0) Big Banana | | | 🞏 (0) Other | | |
|  | Little Orange | 🞏 (1) Little Orange | | 🞏 (0) Big Orange | | | 🞏 (0) Other | | |
|  | | | | | | | | | |
| **Categorisation**  **Ducks & Buckets** | **Trial Error** | 🞏 (97) Examiner 🞏 (98) Child B 🞏 (99) Child L | | | | | | | |
|  | **Trial** |  | | | | **(1) Correct** | | **(0) Incorrect** | |
|  | Rule Check | Can you show me where big ducks go? | | | | 🞏 (1) BIG | | 🞏 (0) Little | |
|  | Rule Check | Can you show me where little ducks go? | | | | 🞏 (1) Little | | 🞏 (0) BIG | |
|  | 1 | Here is a LITTLE duck. | | | | 🞏 (1) little | | 🞏 (0) BIG | |
|  | 2 | Here is a BIG duck. | | | | 🞏 (1) BIG | | 🞏 (0) Little | |
|  | 3 | Here is a LITTLE duck. | | | | 🞏 (1) little | | 🞏 (0) BIG | |
|  | 4 | Here is a BIG duck. | | | | 🞏 (1) BIG | | 🞏 (0) Little | |
|  | 5 | Here is a BIG duck. | | | | 🞏 (1) BIG | | 🞏 (0) Little | |
|  | 6 | Here is a LITTLE duck. | | | | 🞏 (1) little | | 🞏 (0) BIG | |
| **MUST CATEGORISE 5 OUT OF 6 CORRECTLY TO CONTINUE** | | | | | | | | | |
| **Reverse Categorisation**  **Ducks & Buckets** | **Trial Error** | 🞏 (97) Examiner 🞏 (98) Child B 🞏 (99) Child L | | | | | | | |
|  | **Trial** |  | | | | **(1) Correct** | | **(0) Incorrect** | |
|  | Rule Check | Where big ducks go in silly game? | | | | 🞏 (1) Little | | 🞏 (0) BIG | |
|  | Rule Check | Where little ducks go in silly game? | | | | 🞏 (1) BIG | | 🞏 (0) Little | |
|  |  | Here is a LITTLE duck. | | | | 🞏 (1) BIG | | 🞏 (0) Little | |
|  |  | Here is a BIG duck. | | | | 🞏 (1) Little | | 🞏 (0) BIG | |
|  |  | Here is a LITTLE duck. | | | | 🞏 (1) BIG | | 🞏 (0) Little | |
|  |  | Here is a LITTLE duck. | | | | 🞏 (1) BIG | | 🞏 (0) Little | |
|  |  | Here is a BIG duck. | | | | 🞏 (1) Little | | 🞏 (0) BIG | |
|  |  | Here is a BIG duck. | | | | 🞏 (1) Little | | 🞏 (0) BIG | |
|  | | | | | | | | | |

|  | | | | | | | |
| --- | --- | --- | --- | --- | --- | --- | --- |
| **Multi-Search Multi-Location Task**  **PRE-SWITCH** | **Trial Error** | 🞏 (97) Examiner 🞏 (98) Child B 🞏 (99) Child L | | | | | |
|  | Trial | Black Diamond | No Response  98 or 99 | Full Credit  (2) | | Part Credit  (1) | No Credit  (0) |
|  | Training | 1 | □ | □ | | □ | □ |
|  | Training | 2 | □ | □ | | □ | □ |
|  | Training | 3 | □ | □ | | □ | □ |
|  | **Pre-Switch**  **Trials** | No Response  98 or 99 | | Yellow Circle  Error (0) | | **Blue Triangle**  **Correct (1)** | Green Square  Error (0) |
|  | 1 | □ | | □ | | **□** | □ |
|  | 2 | □ | | □ | | **□** | □ |
|  | 3 | □ | | □ | | **□** | □ |
|  | 4 | □ | | □ | | **□** | □ |
|  | 5 | □ | | □ | | **□** | □ |
|  | 6 | □ | | □ | | **□** | □ |
| **MUST HAVE 3 CONSECUTIVE CORRECT RESPONSES TO CONTINUE** | | | | | | | |
| **Multi-Search Multi-Location Task**  **POST-SWITCH** | **Post Switch Trials** | No Response  98 or 99 | Yellow Circle  Non-Perseverative Error (0) | | Blue Triangle Perseverative  Error (0) | | **Green Square Correct (1)** |
|  | 1 | □ | □ | | **□** | | □ |
|  | 2 | □ | □ | | **□** | | □ |
|  | 3 | □ | □ | | **□** | | □ |
|  | 4 | □ | □ | | **□** | | □ |
|  | 5 | □ | □ | | **□** | | □ |
|  | 6 | □ | □ | | **□** | | □ |
|  | 7 | □ | □ | | **□** | | □ |
|  | 8 | □ | □ | | **□** | | □ |

| **NOTES:** |
| --- |
|  |
|  |
|  |
|  |
|  |
|  |
|  |

**SNACK DELAY**

| Examiner error during trial makes trial unscorable 🞏 (97) |
| --- |
| Child refused to do whole task – Behaviour problem 🞏 (98) |
| Child didn’t understand task—Language 🞏 (99) |

**Introduction:**

Check with parent as to acceptable treat. Confirm what child would call it e.g. M&Ms may be lollies. ***Tick the treat used.***

Fruit Loops_____ M&Ms _____ Cheerios _____ Raisins ______ Popcorn_____ Other______

**I have a special treat for you!**

**Warm-up (no delay):**

Place black mat on table---Place treat under clear cup on top of black mat)

**E:** **I have this yummy** *(name treat)*_**for you! You can have it. Get the** *( name treat)*_.

Practice 1 🞏 **Retrieved snack**

🞏 No retrieval (explain) ____________________________

______________________________________________

**Mmm. That’s yummy, huh?** *(allow Child to eat treat)*

**Let’s do it again.** *(place another treat under cup)*

**You can have it. Get the** *( name treat)*_.

Practice 2 🞏 **Retrieved snack**

🞏 No retrieval (explain) ____________________________

______________________________________________

**That’s fun, huh?**

**SNACK DELAY Trials:**

**Examiner:** **OK. Now we’re going to play a game. I have some more (***name treat***) for you!**

(place bell on table for remainder of task)

**But you have to wait until I ring this bell** *(ring bell)* **before you can go get it. Don’t get the (***name treat****)***  (*shake head*) **until I ring the bell.**

**OK. Let’s try it. Remember, you have to wait until I ring this bell before you can go get it. Don’t get it until you hear the bell.** *(Say before each trial)*

Place treat under cup with one hand, begin timing immediately with timer in other hand, place free hand above bell throughout waiting period; say **OK** after each trial, then give reminder--**Remember, you have to wait until I ring this bell before you can go get it. Don’t get it until you hear the bell.** Repeat for each trial. Stop trials after **one** failure.

| **Trial** | **Time**  **(seconds)** | **R T**  **(seconds)** | **Full Wait**  **Successful Trial** | **Partial**  **Success** | **Failed**  **Trial** | **Retrieved the Treat** |
| --- | --- | --- | --- | --- | --- | --- |
| **1** | **5** |  | 🞏 | 🞏 | 🞏 | 🞏 (0)No 🞏 (1) Yes |
| **2** | **15** |  | 🞏 | 🞏 | 🞏 | 🞏 (0)No 🞏 (1) Yes |
| **3** | **30** |  | 🞏 | 🞏 | 🞏 | 🞏 (0)No 🞏 (1) Yes |
| **4** | **45** |  | 🞏 | 🞏 | 🞏 | 🞏 (0)No 🞏 (1) Yes |

**Directions for Scoring:**

1. *Record RT and tick only one trial outcome. =* **Full Wait-Successful trial, Partial Success,** *or* **Failed Trial**
2. *Wait 30 seconds for child to retrieve treat and eat it. If child doesn’t retrieve the treat put a tick “No” in* **Retrieved the Treat** *column and move on to next trial. If child retrieves the treat, wait for the child to eat it before you move to next trial or task.*

**Response Time (RT)** = Time delay from end of wait period (5, 15, 30 or 45) to retrieving treat or child touching treat. Record the number of seconds from when bell rings and child picks up treat.

**Full Wait – Successful Trial** = Child waits to retrieve treat without touching the glass.

**Partial Success** = Child does not retrieve treat but touches the glass during the wait period.

**Failed Trial** = (1) Child retrieving the treat before the bell is rung; or (2) **child** ringing the bell.

**END TRIALS AFTER CHILD FAILS ONE TRIAL.**

**FRUIT STROOP TASK**

| Examiner error during trial makes trial unscorable 🞏 (97) |
| --- |
| Child refused to do whole task – Behaviour problem 🞏 (98) |
| Child didn’t undrstand task—Language 🞏 (99) |

**Look here!** *Place BIG apple, orange, and banana in a row in front of Child---then LITTLE apple, orange, and banana below them. (Place pictures starting at Child’s left)*

**I have a BIG APPLE** (big voice) **and a LITTLE APPLE** (little voice).

**A BIG ORANGE** (big voice) **and a LITTLE ORANGE** (little voice)

**And a BIG BANANA** (big voice) **and a LITTLE BANANA** (little voice).

*Remove the row of little pictures and ask child to identify large pictures of fruit.*

**Identification:**

*Correct child if he/she does not point to the correct picture. Tick child’s response.*

1. **Show me the APPLE 🞏 Apple 🞏** Other
2. **Show me the ORANGE** 🞏 **Orange** 🞏 Other

1. **Show me the BANANA** 🞏 **Banana** 🞏 Other

**OK!** *Remove row of big pictures---place embedded fruit cards in front of Child – large banana, large apple, large orange - left to right and begin Stroop Trials.*

**Stroop Trials:**

*Provide no verbal feedback---tick picture child points to.*

1. **Now show me the LITTLE apple**

**🞏 Little apple** 🞏 Big apple 🞏 Other

1. **Now show me the LITTLE banana**

🞏 **Little banana** 🞏 Big banana 🞏 Other

1. **Now show me the LITTLE orange**

🞏 **Little Orange** 🞏 Big Orange 🞏 Other

**DUCKS AND BUCKETS--CATERGORISATION**

| Examiner error during trial makes trial unscorable 🞏 (97) |
| --- |
| Child refused to do whole task – Behaviour problem 🞏 (98) |
| Child didn’t understand task—Language 🞏 (99) |

**Categorisation Training:**

Introduction of Stimuli:

Use different voices for big ducks and buckets (regular voice) and little ducks and buckets (higher pitch) throughout.

**Look what I have here! I have a big duck, and a little duck.**

*(show child big and little blue ducks)*

**Practice Trials:**

**Now, I have these buckets here.** (*Place big bucket on child’s left and little bucket on child’s right.*

*Place buckets approximately 20cm from child’s edge of table and approximately 10cm apart*.)

**I’m going to put the big ducks in the big buckets and the little ducks in the little bucket.** *(pointing to each bucket, slowly)*.

**Here’s a big duck. It goes in the big bucket.** *(put it in)*

**Here’s a little duck. It goes in the little bucket.** *(put it in)*

**Now it’s your turn to put the big ducks in the big bucket and the little ducks in the little bucket.**

**Rule Check:**

1. **Can you show me where the big ducks go?**

*If Correct:* **Very good, that’s right.** Then proceed to Rule Check 2.

If Incorrect: **Uh oh. Remember, in this game, all the big ducks go in the big bucket and all the little ducks go in the little buckets.** Point to appropriate buckets. Tick response.

**Response:** 🞏 **Correct (1)** 🞏 **Incorrect (0)**

1. **Can you show me where the little ducks go?**

If Correct: **Very good, that’s right.** Then go to**… Okay, so let’s put all of these ducks in their buckets.**

If Incorrect: **Uh oh. Remember, in this game, all the big ducks go in the big bucket and all the little ducks go in the little buckets.** (Point to appropriate buckets). Tick response.

**Response:** 🞏 **Correct (1)** 🞏 **Incorrect (0)**

**Okay, so let’s put all of these ducks in their buckets.**

**Categorisation Trials: Duck Sorting**

1. *If child doesn’t like you repeating the rules on each trial, say* **“I have to say it every time, it’s the rule.”**
2. *If the child points to the bucket Experimenter may sort the duck for him/her.*
3. *Do not say* ***“okay”*** *in response to sort. Say***, “Let’s do another one,” “Let’s do it again,”** *etc.*
4. *If child asks for help, respond* **“whatever you think”,** **“you choose”,** *etc.*
5. *Point to buckets by touching the top with a finger. Do not bring out the duck until the rule statement is complete. (e.g. Big ducks go here, little ducks go here-*🡪 *here is a big duck (while simultaneously bringing out big duck)*
6. *Once duck has been presented, Experimenter CANNOT repeat the rule.*
7. *Say the identifying statement while simultaneously displaying the duck.*
8. *Present ducks above and between buckets.*

On *each* trial, say: **If it is a big duck, then put it here, but if it is a little duck, put it here.**

Tick big or little bucket that child puts duck in on each trial

| Trial |  | **(1) Correct Responses** | **(0) Incorrect Responses** |
| --- | --- | --- | --- |
| 1 | **Here is a LITTLE duck.** | **🞏 little** | 🞏 BIG |
| 2 | **Here is a BIG duck.** | **🞏 BIG** | 🞏 little |
| 3 | **Here is a LITTLE duck.** | **🞏 little** | 🞏 BIG |
| 4 | **Here is a BIG duck.** | **🞏 BIG** | 🞏 little |
| 5 | **Here is a BIG duck.** | **🞏 BIG** | 🞏 little |
| 6 | **Here is a LITTLE duck.** | **🞏 little** | 🞏 BIG |
|  | ***Response Totals*** |  |  |

**GO ON TO REVERSE CATEGORISATION if child gets at least 5 out of 6 correct. If child DOES NOT get 5 out of 6 correct GO TO MULTILOCATION TASK.**

**DUCKS AND BUCKETS—REVERSE CATEGORISATION**

| Examiner error during trial makes trial unscorable 🞏 (97) |
| --- |
| Child refused to do whole task – Behaviour problem 🞏 (98) |
| Child didn’t understand task—Language 🞏 (99) |

**OK, now let’s play a “silly” game. Let’s put all the big ducks in the little bucket** *(point, slowly)* **and put all the little ducks in the big bucket** *(point, slowly)*. **This is a silly game**.

**Rule Check:**

**1. Can you show me where the big ducks go in this silly game?**

If Correct: **Very good, that’s right.**

If Incorrect: **Uh oh. Remember, in this silly game, all the big ducks go in the little bucket and all the little ducks go in the big bucket.** Tick response.

**Response:** 🞏 (1) **Correct** 🞏 (0) **Incorrect**

**2. Can you show me where the little ducks go in this silly game?**

If Correct: **Very good, that’s right.**

If Incorrect: **Uh oh. Remember, in this silly game, all the big ducks go in the little bucket and all the little ducks go in the big buckets.** Tick response.

**Response:** 🞏 (1) **Correct** 🞏 (0) **Incorrect**

**Okay, let’s try this silly game!**

**Reverse Categorisation Trials:**

1. *If the child points to the bucket Examiner may sort the duck for him/her.*
2. *Do not say “okay” in response to sort. Say,* **“Let’s do another one,” “Let’s do it again,”** *etc.*
3. *If child asks for help, respond* **“whatever you think”,** **“you choose”,** *etc.*
4. *Point to buckets by touching the top with a finger. Do not bring out the duck until the rule statement is complete. (Example***: Big ducks go here, little ducks go here***-*🡪 **here is a big duck** *while simultaneously bringing out big duck).*
5. *Once duck has been presented, Experimenter CANNOT repeat the rule.*
6. Say the identifying statement while simultaneously displaying the duck.
7. Present ducks above and between buckets.

*On each trial, say:* **If it is a big duck, then put it here, but if it is a little duck, put it here.** *Tick big or little bucket that child puts duck in on each trial.*

| Trial |  | (1) Correct Responses | (0) Incorrect Responses |
| --- | --- | --- | --- |
| 1 | **Here is a LITTLE duck.** | **🞏 BIG** | 🞏 little |
| 2 | **Here is a BIG duck.** | **🞏 little** | 🞏 BIG |
| 3 | **Here is a LITTLE duck.** | **🞏 BIG** | 🞏 little |
| 4 | **Here is a LITTLE duck.** | **🞏 BIG** | 🞏 little |
| 5 | **Here is a BIG duck.** | **🞏 little** | 🞏 BIG |
| 6 | **Here is a BIG duck.** | **🞏 little** | 🞏 BIG |
| ***Response Totals*** | |  |  |

**MULTI-SEARCH MULTI-LOCATION**

| Examiner error during trial makes trial unscorable 🞏 (97) |
| --- |
| Child refused to do whole task – Behaviour problem 🞏 (98) |
| Child didn’t understand task—Language 🞏 (99) |

**Materials:**

1. Testing apparatus
2. Treats
3. Scoring sheets
4. Stopwatch

**Introduction:**

Check with parent as to acceptable treat. Confirm what child would call it e.g. M&Ms may be lollies. Tick the treat used.

Fruit Loops _____M&Ms _____ Cheerios _____ Raisins ______ Popcorn_____ Other_____

**Training:**

**Look what I have here!**

1. Examiner presents apparatus within reach of child with only the black diamond shape attached to the center drawer.
2. Examiner demonstrates the following steps a) lifting the felt cover, b) pulling on the black diamond, and c) revealing the treat. When treat is revealed say…**There’s a (**name treat**) in there. You can have it.**
3. Examiner allows child to retrieve treat---then slides apparatus slightly away from the child back towards examiner and completes the following steps while child is attending: a) places new treat in center drawer; b) closes the drawer; c) covers the apparatus with the black felt cover; d) pushes apparatus to within child’s easy reach, and says…**Can you find the (**name treat**)?**
4. Record highest credit achieved over three trials:

**1 2 3**

🞏 🞏 🞏 (2) Full credit = removes cover, pulls black diamond, retrieves treat without assistance.

**1 2 3**

🞏 🞏 🞏 (1) Partial credit = completed some but not all steps. Explain ___________________________

________________________________________________________________________________

**1 2 3**

🞏 🞏 🞏 (0) No credit = child did not complete any of the steps. Explain__________________________

________________________________________________________________________________

1. Repeat step 3 if necessary, with modeling, up to **3** times.
2. Prepare apparatus for trials…. Say… **Alright!** Then move the apparatus out of child’s reach, remove the black diamond shape on middle drawer, and position the yellow circle, blue triangle, and green square onto the drawers in order starting from Child’s left to right.

**Test--Pre-switch Directions:**

1. Hold treat above blue triangle drawer until Child is attending---then place it in drawer and say…**Here’s the (**name treat) while pointing to the blue triangle.
2. Point to the yellow circle attached to the drawer and say… **There’s no (**name treat**) here.**
3. Point to the green square attached to the drawer and say… **There’s no (**name treat**) here.**
4. Return to blue triangle drawer, point to blue triangle and say… **Here’s the (**name treat**).**
5. Slide the apparatus back towards Examiner out of Child’s reach and cover with felt cover.
6. Once the cover is in place slide the apparatus back towards Child and say… **Can you find the (**name treat **)?** Allow up to 30 sec for response. If child does not choose a drawer, then repeat instructions. Allow only 1 attempt to choose the correct drawer. If they open an incorrect drawer before choosing the correct drawer put a tick in their first choice. If they do not respond at all put “ 98” or “99” in No Response column.
7. Record result by placing a tick in appropriate column in the result box below---repeat until achieves **3** consecutive correct trials at the blue triangle location.

| Trial | Yellow Circle | **Blue Triangle** | Green Square | No Response |
| --- | --- | --- | --- | --- |
| **1** |  |  |  |  |
| **2** |  |  |  |  |
| **3** |  |  |  |  |
| 4 |  |  |  |  |
| 5 |  |  |  |  |
| 6 |  |  |  |  |
| **Total Trials:** |  |  |  |  |

**IF CHILD DOES NOT REACH CRITERION BY THE END OF 6 TRIALS DO NOT GO ON TO POST-SWITCH.**

**Test: Post-switch Directions:**

**Now we are going to play a silly game. Watch what I do!**

1. Hold treat above the green square drawer until Child is attending, then place it in the drawer. Say...**Here’s the (**name treat**)** while pointing to the green square.
2. Point to yellow circle attached to drawer saying… **There’s no (**name treat**) here.**
3. Point to blue triangle attached to drawer saying…**There’s no (**name treat**) here.**
4. Return to green square attached to drawer saying… **Here’s the (**name treat**).**
5. Pull apparatus slightly away from child’s reach toward’s examiner. Slowly cover the apparatus with felt allowing a 10 second delay, before sliding the apparatus back towards the child and saying… **Can you find the (**name treat**)?** Allow up to 30 sec for response. If child does not choose a drawer, then repeat instructions. Allow only 1 attempt to choose the correct drawer. If they open an incorrect drawer before choosing the correct drawer put a tick in their first choice. If they do not respond at all put “ 98” or “99” in No Response column.
6. Record result by placing a tick in appropriate column in the result box.
7. Repeat until **2** consecutive correct trials—Child chooses green square first.

| Trial | Response  Time | Yellow Circle  Non-perseverative Error | Blue Triangle  Perseverative Error | **Green Square**  **Correct** | No Response |
| --- | --- | --- | --- | --- | --- |
| **1** |  |  |  |  |  |
| **2** |  |  |  |  |  |
| 3 |  |  |  |  |  |
| 4 |  |  |  |  |  |
| 5 |  |  |  |  |  |
| 6 |  |  |  |  |  |
| 7 |  |  |  |  |  |
| 8 |  |  |  |  |  |
|  |  | Total: | Total: | Total: | Total: |
